# Linkages between health and agriculture sectors in Ethiopia: a formative research study exploring barriers, facilitators and opportunities for local level coordination to deliver nutritional programmes and services

**DOI:** 10.1186/s40795-017-0189-4

**Published:** 2017-08-02

**Authors:** Girmay Ayana, Tesfaye Hailu, Desalegn Kuche, Andinet Abera, Solomon Eshetu, Alemnesh Petros, Aweke Kebede, Masresha Tessema, Cami M. Allen, Mihretab M. Salasibew, Alan D. Dangour

**Affiliations:** 1 Ethiopian Public Health Institute, Addis Ababa, Ethiopia; 2 London School of Hygiene & Tropical Medicine, London, UK

**Keywords:** Agriculture, Undernutrition, Coordination, Multi-sectoral, Linkage, Ethiopia

## Abstract

**Background:**

In Ethiopia, poor infant and young child feeding practices and low household dietary diversity remain widespread. The Government has adopted the National Nutrition Programme that emphasizes the need for multi-sectoral collaboration to effectively deliver nutrition-sensitive and nutrition-specific interventions. The Sustainable Undernutrition Reduction in Ethiopia (SURE) programme is one such Government-led initiative that will be implemented jointly by the health and agriculture sectors across 150 districts in Ethiopia. Prior to the design of the SURE programme, this formative research study was conducted to understand how the governance structure and linkages between health and agriculture sectors at local levels can support implementation of programme activities.

**Methods:**

Data were collected from eight districts in Ethiopia using 16 key informant interviews and eight focus group discussions conducted with district and community-level focal persons for nutrition including health and agriculture extension workers. A framework analysis approach was used to analyze data.

**Results:**

Few respondents were aware of the National Nutrition Programme or of their own roles within the multi-sectoral coordination mechanism outlined by the government to deliver nutritional programmes and services. Lack of knowledge or commitment to nutrition, lack of resources and presence of competing priorities within individual sectors were identified as barriers to effective coordination between health and agriculture sectors. Strong central commitment to nutrition, increased involvement of other partners in nutrition and the presence of community development workers such as health and agriculture extension workers were identified as facilitators of effective coordination.

**Conclusions:**

Federal guidelines to implement the Ethiopian National Nutrition Programme have yet to be translated to district or community level administrative structures. Sustained political commitment and provision of resources will be necessary to achieve effective inter-sectoral collaboration to deliver nutritional services. The health and agriculture extension platforms may be used to link interventions for sustained nutrition impact.

## Background

Ethiopia is primarily an agrarian society. The economy relies heavily on rain-fed agriculture, employing 76% of the workforce and accounting for 41% of the national GDP [1]. Ethiopia has diverse agro-climatic environments and produces a variety of foods, but child dietary diversity remains poor [2]. Undernutrition remains prevalent and only 7% of children 6–23 months consume the minimum acceptable diet [3]. A comparative risk assessment in Ethiopia reported that childhood wasting, underweight and stunting were major risk factors for deaths due to diarrhoeal diseases and other common infections among children under-five years of age [4].

Agriculture is a major livelihood for nutritionally vulnerable people in developing countries [5]. Advances in agriculture can provide more and better quality food to improve nutritional status of individuals but there is often a disconnect between improved agricultural practices and nutritional outcomes at the household level [6]. Promotion of nutrition-sensitive agriculture can contribute to improved dietary diversity and increased income (from agricultural activities) can facilitate the purchase of nutritious food and health services [7]. However, intra-sectoral political commitment to nutrition-sensitive approaches is often limited in agriculture [8].

Ethiopia has a decentralized platform of community-based nutrition service delivery with an emphasis on district and local level management [9]. The health platform comprises trained female health extension workers who provide basic primary health care service at the community level [10]. Agriculture services have historically comprised an important component of the government’s development agenda, and the extension platform includes workers who provide demonstration services and technical support to transfer knowledge and skills [11]. Health and agriculture extension workers are constituent members of the community-level multi-sectoral nutrition coordination committee.

The Ethiopian National Nutrition Programme provides a framework for multi-sectoral collaboration to effectively deliver nutrition interventions (both nutrition-specific and nutrition-sensitive) [9]. To implement the programme, federal guidelines on the structure, role and function of multi-sectoral nutrition coordination mechanisms have also been developed [12]. The National Nutrition Coordination Body and technical committees oversee central level coordination, while similar structures are mandated at regional, district and community levels. The routine function of the committees is monitored through reporting systems and supportive supervision.

The Sustainable Undernutrition Reduction in Ethiopia (SURE) programme is a government-led intervention that will be implemented in 150 districts from 2017 to 2019. The programme aims to reduce stunting and to increase prevalence of minimum acceptable diet through counselling on child feeding practices and dietary diversity to be jointly delivered to caregivers by health and agriculture extension workers. SURE also aims to strengthen multi-sectoral coordination committees at district and community levels.

This formative research study was conducted to understand how governance structures and linkages between the health and agriculture sectors might support implementation of programme activities at local levels. Findings from this study are intended to inform the design of the SURE programme and other integrated interventions that depend on effective multi-sectoral coordination for nutrition.

## Methods

### Study aim

This aim of this qualitative study was to identify barriers, facilitators and opportunities for local level coordination between health and agriculture sectors to deliver nutritional services in Ethiopia.

### Study setting

This study was conducted in August 2015 in four agrarian regions of Ethiopia: Oromiya, Amhara, SNNP and Tigray regions.

### Study sample selection

**Fig. 1:**
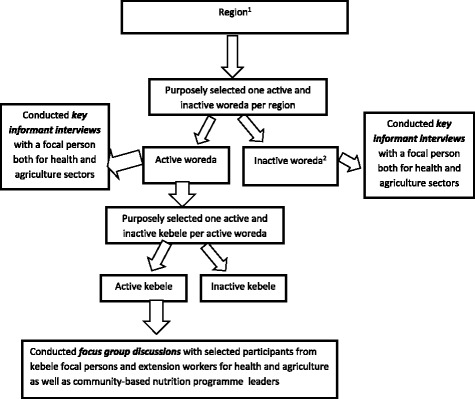
Flow chart showing the selection process of participants per region and data collection activities. ^1^The same process was applied to all four regions namely: Oromiya, Ahmara, SNNP and Tigray. ^2^ No participants were selected from kebeles under the inactive woreda

All woreda (districts) and kebeles (smallest administrative units) in the four regions were eligible for inclusion and samples were selected using purposive sampling method. In order to ensure heterogeneity of study participants, we selected woreda and kebeles according to the known presence or absence of effective multi-sectoral coordination committees. Those with functional multi-sectoral coordination committees in place were designated ***active sites*** and those without were designated ***inactive sites*** (Fig. 1). These classifications were based on locally available performance reports obtained from the regions about multi-sectoral coordination activities at district and kebele levels. A functional multi-sectoral coordination committee was defined as a committee composed of representatives of all 9 relevant sectors and actively conducting activites as demonstrated by joint planning, regular meetings based on a set of agenda and record of providing supportive supervision to the provision of nutritional services in the community. There were 16 woreda (8 ***active*** and 8 ***inactive***) and 8 kebeles (4 ***active*** and 4 ***inactive***) selected across the four regions.

Study participants represented various institutions listed below:

- Sectoral focal points for health and agriculture
- Focal point for Community-Based Nutrition programme (CBN), a set of nutrition-sensitive interventions delivered to women and children under 2 years by the Health Extension Platform
- Focal point for Agriculture Extension Programme
- Other health/agriculture district officials
- Health and agriculture extension workers and community leaders

### Data collection

Data was collected using 16 key informant interviews (KIIs) at woreda level and 8 focus group discussions (FGDs) at kebele level. Participants were asked about existing functionality of local level multi-sectoral coordination and about barriers, facilitators and future opportunities for effective multi-sectoral coordination to deliver integrated nutrition services to the community. The number of interviews and focus group discussions depended upon reaching a theoretical saturation point in each category of ***active*** and ***inactive*** districts and kebeles.

Data collectors were selected using pre-defined criteria by their educational background and previous experience on qualitative data collection. They were given 4 days of training by investigators at the Ethiopian Public Health Institute (EPHI) on principles of qualitative research, the study topic guide, and interviewing and facilitation skills. As part of their training, data collectors were involved in pilot testing and also given in-field supervision by EPHI staff. Interviews and group discussions were conducted using local languages. Prior to interview data collectors arranged separate, conducive rooms. Key informant interviews were conducted for about 30 min and group discussions lasted on average 60 min. All sessions were recorded using digital audio recorders. Audio recordings were kept in a safe lockable cabinet while on field and transferred to the head office at EPHI and only the principal investigator had access to the data.

### Data analysis

Audio recordings were transcribed by EPHI staff. Specific themes were identified and coded. We identified facilitators using data from the active districts and kebeles with functioning coordination mechanisms, whereas the barriers were identified from inactive sites.

Framework analysis approach was used to analyze collected qualitative data. The five stages of framework analysis approach were followed: 1) familiarization (reading through the transcribed data), 2) identifying a thematic framework (start coding), 3) indexing (coding using numerical or textual codes), 4) charting (create charts representing the data) and 5) mapping and interpretation (searching for patterns, concepts, ideas etc.). Qualitative data analysis software Nvivo version 10 was used to code and construct thematic areas from the collected data.

## Results

### Demographic characteristics of participants

In total, there were 45 participants from the agriculture sector and 38 participants from the health sector. At woreda level, there were 16 key informant interviewees, and at kebele level, there were 67 participants in the focus discussions (Table 1).

**Table 1:** Characteristics of study participants

Characteristics	Region				
	Oromiya	SNNP	Amhara	Tigray	Total
Age					
20–30	17	10	17	8	52
31–40	5	8	3	6	22
41–50	0	1	0	3	4
> 50	0	1	1	3	5
Gender					
Male	16	12	13	11	52
Female	6	8	8	9	31
Agriculture sector	12	11	11	11	45
Health sector	10	9	10	9	38
No of KII^a^ participants	4	4	4	4	16
No of FGD^b^ participants	18	16	17	16	67

^a^Key Informant Interviews

^b^Focus Group Discussions

### Facilitors to effective multi-sectoral coordination

In the active woreda and kebeles, there was evidence of an established and functioning nutrition coordination committee. Participants reported regular participation in an established local nutrition committee represented by multiple sectors, and demonstrated awareness of the National Nutrition Programme and of their responsibilities under the subsequent guideline for multi-sectoral coordination.

*“Nutrition is an issue for all of us in this kebele and we are aware of the government nutrition programme. We have committee involving various sectors.. It is coordinated by the health sector and agriculture is the deputy coordinator….all sectors have signed a joint work plan and have an agreement to work together” (FGD participant, Agriculture sector)*

Nutrition interventions such as raising awareness about complementary feeding and dietary diversity were delivered to the community using existing government structures and community networks in the active sites, and were supported by the coordination mechanisms.

*“… we gave pictorially supported training to the community on how to prepare food for the children. For example, we showed them how to prepare a soup using the following recipes: 3 cups of water, 1-cup flour, ½ coffee cup of oil all mixed in a bowl. We also trained mothers on how to prepare foods using locally available fruits and vegetables; such as kale, pumpkin, potato and others…we informed mothers that if they prepare diversified food using locally available food items, their children will not be malnuritioned.” (FGD participant, Agriculture sector)*

Strong leadership and commitment to nutrition within the local government structure was the main facilitator to establish and support effective inter-sectoral coordination between the health and agriculture sectors.

*“Yes, there is strong commitment and supportive supervision from the district officials … on the role of committee, timing of the meeting, type of participants and the like.” (FGD participant, Health sector)*

The presence of extension workers who were actively engaged in nutrition also facilitated the successful delivery of nutritional services to the community. A focus group discussion participant from the agriculture sector in an active site described how the committee functions:

*“The government structure itself ties both sectors together. It consists of health extension worker, agriculture development agent, administration personnel and also representatives from the education sector. Therefore, when we meet for a certain issue, the health extension workers are with us to update one another about local nutritional programmes…”*

The presence of community networks with reach at household level and the increasing engagement of development partners in nutrition such as NGOs were also reported as strong facilitators for good health and agriculture linkages at community level.

*“… NGOs support us by working at the grass roots level. They provide us with the necessary training and support our activities, so their contribution to nutrition has been valuable” (FGD participant, Health sector)*

### Barriers to effective multi-sectoral coordination

Lack of nutrition knowledge was cited by participants as a major barrier against establishing effective coordination structures at both woreda and kebele levels. Poor awareness of the nutrition problem and lack of commitment by local government structures hindered effective inter-sectoral collaboration between the health and agriculture sectors.

*“There is lack of awareness on the importance of collaboration among committee members, the health extension workers and agriculture development agent” (Nutrition focal person, agriculture sector)*

In districts with no effective linkages between the health and agriculture sectors (***inactive sites***), participants reported that each sector focused only on its own priorities rather than working in collaboration to achieve local nutrition objectives.

*“There is an established committee but it is poor in supporting sectors to work together. Every sector is running by itself and all sectors are not working in collaboration” (Nutrition focal person, Agriculture sector)*

Other barriers reported were lack of logistics and supply, workload and presence of natural emergencies such as drought, which draw resources away from routine work.

### Opportunities to link service delivery

Participants reported that the presence of professional community-level health and agriculture extension platforms represented a clear opportunity to collaborate to link service delivery for improved community nutrition outcomes. The health and agriculture extension workers who provide key services are well-established professionals supported by volunteer community leaders.

*“We, as health and agriculture extension workers, are responsible for a lot of households in our kebele and deliver various services. We used to travel a long distance before, but currently we have health development armies who are helping us to provide nutrition services nearer to mothers and children in their home” (Health extension worker, FGD participant)*

Health and agriculture sector participants also reported willingness to collaborate to provide nutritional services when aligned with a favorable political environment and increasing government commitment to nutrition.

*“…there is a government guideline from above and we are the implentors. Only the two health extension workers can not do all the job in nutrition. They should rather work in coordination with the kebele administer, agricultural extension workers and health development armes. Therefore, even if the idea is new for me, we are willing to work together…” (Agricultural extension worker, FGD participant)*

Other opportunities to link services include the presence of key infrastructure such as farmer demonstration sites and training centers and other facilities used to show food preparation and gardening.

## Discussion

While functional multi-sectoral committees were identified in some localities, we found that few respondents at local levels of government were aware of the Ethiopian National Nutrition Programme. Facilitators of collaboration included the existence of strong local government commitment to nutrition and the presence of community networks. Barriers of effective coordination included poor awareness of the nutrition problem or of mandated coordination structures, weak political commitment, competing priorities, and lack of resources. Prior investments in community-level health and agriculture platforms and infrastructure provide a key opportunity to accelerate both coordination and the delivery of nutritional services.

There is little evidence to date on multi-sectoral coordination for nutrition [13, 14]. However, consistent with our findings, a study on nutrition governance in Ethiopia that explored the views of actors at central and regional levels also found limited knowledge of the National Nutrition Programme, with large differences reported between sectors in their level of awareness [15]. Of those who were aware of the programme, representatives of non-health sectors were more likely to report the view that their own sectors were not required to contribute to improved nutrition outcomes. For example, representatives from the Federal Ministry of Agriculture perceived the National Nutrition Programme to be a health sector initiative and did not identify a clearly defined role for agriculture [15].

Our results confirm that key challenges to effective coordination of nutrition activities include insufficient political commitment and the presence of competing priorities in non-health sectors. In agriculture, the ability to integrate nutrition and other intra-sectoral aims into a single framework may be poor [16]. Specialists may focus on technical challenges of production and may have poor knowledge outside of their fields of expertise [17]. It will be necessary to develop technical expertise in nutrition within the agriculture sector to adopt nutrition-sensitive agriculture interventions, perhaps through the hiring of nutritionists or through the provision of nutrition training to personnel at all levels including agriculture extension workers [18].

Cascading multi-sectoral nutrition structures from central to local levels will depend on improved awareness, leadership and continued political commitment [17]. Provision of information and tools alone is unlikely to be sufficient and advocacy may be needed to sustain commitment and to improve cohesion and coordination [19]. Identifying “champions” who are empowered to advocate for the nutrition agenda may be essential [15], especially to support changes in the views of staff from sectors that have not traditionally prioritised nutrition-sensitive objectives or interventions. It has also been recommended that systems of incentives and accountability are adopted to mainstream nutrition-sensitive approaches within sectors [20]; successful multi-sectoral coordination structures will also depend on such features [21]. Integration of activities may further depend on good administrative organisation and synchronisation of work schedules [22]. Ultimately improvement in nutrition outcomes will depend upon broader recognition by local level government authorities that undernutrition is a multi-sectoral problem and that sectoral priorities must be reframed to support strong participation both in coordination mechanisms and in the delivery of nutrition-sensitive services [23].

This is the one of the first studies to explore the existence and functioning of nutrition coordination mechanisms at district and community levels. The study benefitted from local participant members of both active and inactive nutrition coordination committees. The findings are not generalizable to all districts or communities and this remains one of the key limitations of the study. However, the results provide context to support the establishment and function of local level committees to govern integrated nutrition interventions such as the SURE programme.

## Conclusion

Collaborations between health and agriculture sectors were not adequate at woreda and kebele levels. Federal guidelines to implement the Ethiopian National Nutrition Programme have yet to be translated to relevant administrative structures. Sustained commitment will be necessary to achieve effective inter-sectoral collaboration to deliver nutritional services. The health and agriculture extension platforms may be used to link interventions for sustained nutrition impact.

## Abbreviations

CBN: Community based nutrition
CIFF: Child Investment Fund Foundation
EPHI: Ethiopian Public Health Institute
FGDs: Focus group discussion
GDP: Gross domestic product
KIIs: Key informant interviews
LSHTM: London School of Hygiene and Tropical Medicine
NGO: Non Governmental Organizations
SNNPR: South nations Nationalities and People Region
SURE: Sustainable Under-nutrition Reduction in Ethiopia
